# Supplementary material for: Mapping Soil-Transmitted Helminth Parasite Infection in Rwanda: Estimating Endemicity and Identifying At-Risk Populations
Source: Trop Med Infect Dis. 2019 Jun 14;4(2):93. doi: 10.3390/tropicalmed4020093 (PMC6630518; doi:10.3390/tropicalmed4020093)
Supplement: Supplementary file 1 [file tropicalmed-04-00093-s001.pdf]

Mapping soil-transmitted helminth parasite infection in Rwanda: estimating endemicity and identifying at-risk populations

Supplementary File 1

Eugene Ruberanziza[1*], Kei Owada[2,3*], Nicholas J. Clark[2,3], Irenee Umulisa[1], Giuseppina Ortu[4], Warren Lancaster[5], Tharcisse Munyaneza[6], Aimable Mbituyumuremyi[7], Ursin Bayisenge[1], Alan Fenwick[4], Ricardo J. Soares Magalhães[2,3]

[1] Neglected Tropical Diseases and Other Parasitic Diseases Unit, Malaria and Other Parasitic Diseases Division, Rwanda Biomedical Center, Ministry of Health, Kigali, Rwanda
[2] UQ Spatial Epidemiology Laboratory, School of Veterinary Science, the University of Queensland, Gatton 4343, Queensland, Australia
[3] Children Health and Environment Program, Child Health Research Centre, The University of Queensland, South Brisbane 4101, Queensland, Australia
[4] Schistosomiasis Control Initiative (SCI), Department of Infectious Diseases Epidemiology, Imperial College, London, United Kingdom
[5] The END Fund, New York, New York, United States of America
[6] Microbiology Unit, National Reference Laboratory (NRL) Division, Rwanda Biomedical Center, Ministry of Health, Kigali, Rwanda
[7] Malaria and Other Parasitic Diseases Division, Rwanda Biomedical Center, Ministry of Health, Kigali, Rwanda

Figure S1: Semivariograms of observed and residual spatial dependence.

	**Observed**	**Residual**
Panel A:<br><br>***A. lumbricoides***	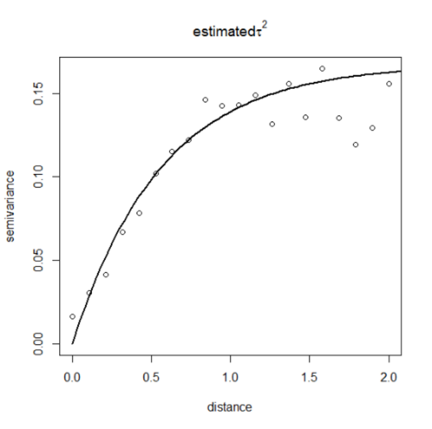	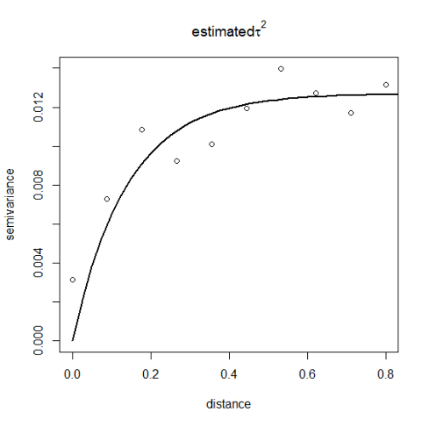
Panel B:<br><br>***T. trichiura***	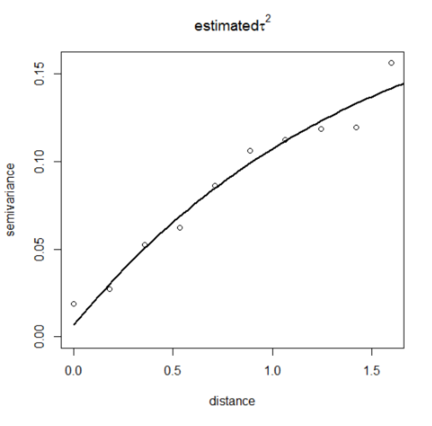	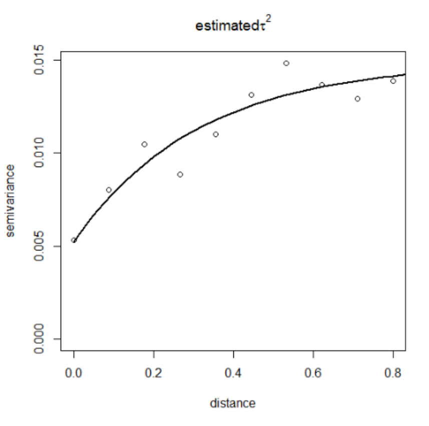
Panel C:<br><br>***Hookworm***	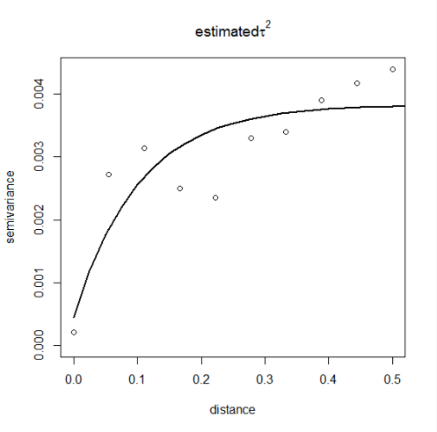	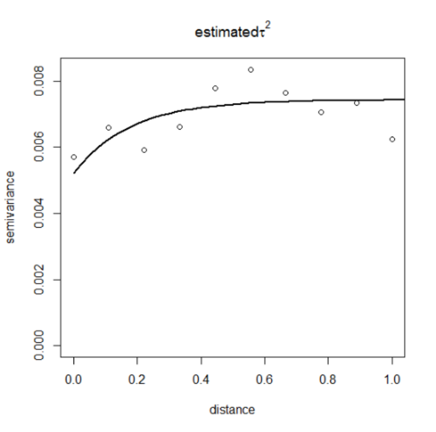

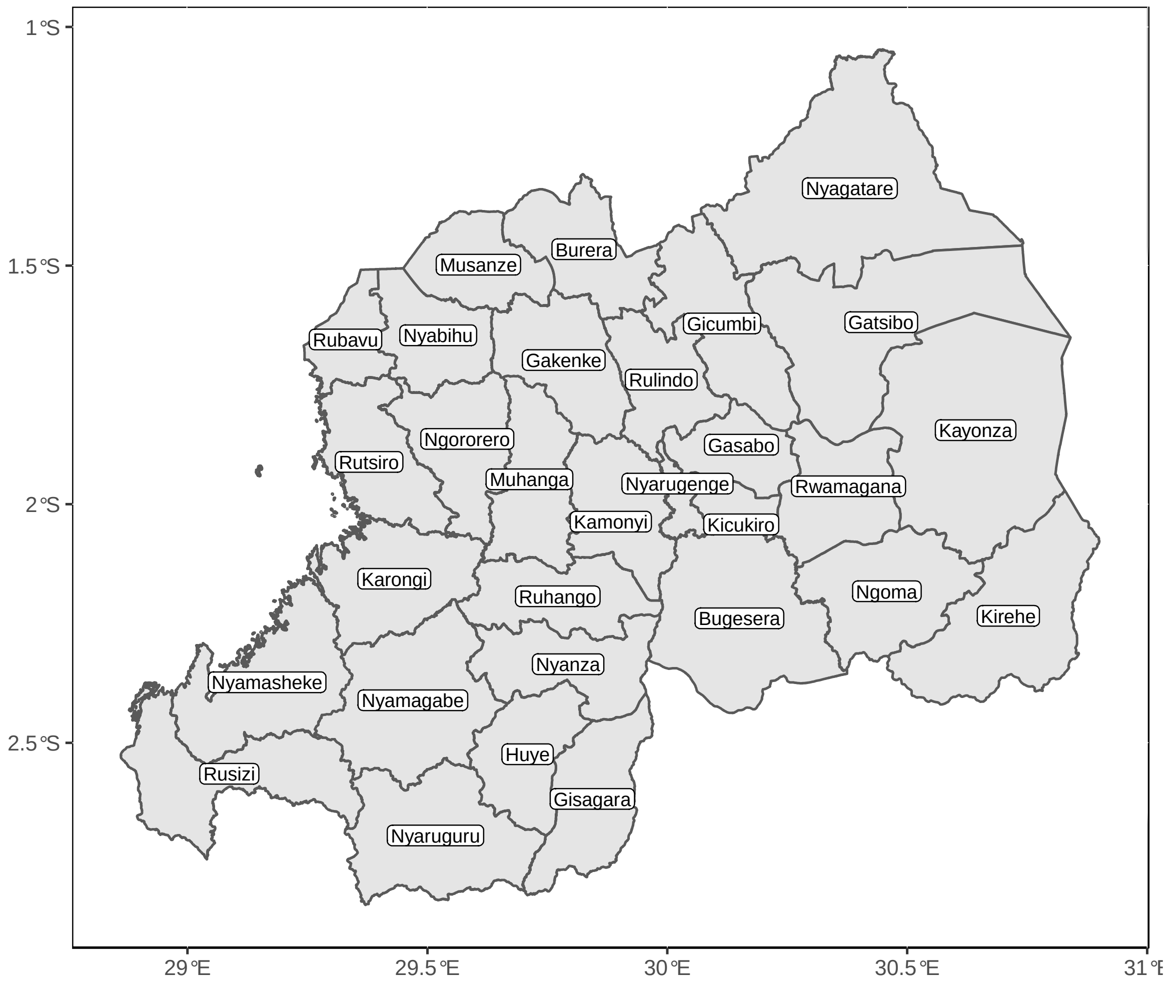

Figure S2: Map of Rwanda's provencial districts. This figure was produced in R version 3.5 using a shapefile representing Rwanda's current administrative units (obtained from the data warehouse DIVA GIS (www.diva-gis.org/Data)).

Table S1: Prevalence of soil-transmitted (STH) helminth infection, represented as proportions of surveyed individuals found to be infected with at least one STH parasite (STH), *A. lumbricoides*, *T. trichiura* and hookworm, in Rwanda across two survey periods

District	2008 STH	2008 Ascaris	2008 Hookworm	2008 Trichuris		2014 STH	2014 Ascaris	2014 Hookworm	2014 Trichuris
Bugesera	0.432	0.028	0.402	0.036		0.108	0.064	0.028	0.036
Burera	0.958	0.865	0.015	0.663		0.802	0.734	0.046	0.542
Gakenke	0.678	0.496	0.347	0.373		0.469	0.403	0.071	0.183
Gasabo	0.152	0.052	0.100	0.013		0.057	0.029	0.017	0.014
Gatsibo	0.543	0.078	0.519	0.053		0.244	0.180	0.048	0.052
Gicumbi	0.686	0.322	0.462	0.136		0.526	0.486	0.011	0.223
Gisagara	0.588	0.279	0.467	0.067		0.293	0.240	0.047	0.037
Huye	0.720	0.614	0.305	0.127		0.455	0.439	0.016	0.053
Kamonyi	0.421	0.100	0.342	0.038		0.196	0.128	0.028	0.048
Karongi	0.675	0.542	0.136	0.301		0.723	0.589	0.031	0.374
Kayonza	0.452	0.013	0.431	0.016		0.177	0.067	0.080	0.063
Kicukiro	0.076	0.040	0.000	0.049		0.021	0.008	0.008	0.004
Kirehe	0.796	0.008	0.779	0.088		0.168	0.040	0.100	0.040
Muhanga	0.753	0.300	0.563	0.057		0.250	0.193	0.043	0.027

District	2008 STH	2008 Ascaris	2008 Hookworm	2008 Trichuris		2014 STH	2014 Ascaris	2014 Hookworm	2014 Trichuris
Musanze	0.947	0.795	0.210	0.705		0.771	0.655	0.033	0.552
Ngoma	0.533	0.004	0.521	0.050		0.105	0.095	0.007	0.003
Ngororero	0.789	0.620	0.413	0.112		0.588	0.508	0.064	0.124
Nyabihu	0.915	0.847	0.119	0.617		0.884	0.824	0.004	0.424
Nyagatare	0.657	0.240	0.492	0.157		0.225	0.053	0.143	0.073
Nyamagabe	0.822	0.744	0.157	0.467		0.563	0.535	0.020	0.220
Nyamasheke	0.922	0.611	0.336	0.725		0.563	0.417	0.051	0.257
Nyanza	0.494	0.085	0.464	0.055		0.128	0.080	0.032	0.020
Nyarugenge	0.203	0.031	0.114	0.095		0.060	0.052	0.000	0.012
Nyaruguru	0.874	0.824	0.167	0.531		0.799	0.779	0.028	0.225
Rubavu	0.955	0.827	0.000	0.909		0.896	0.779	0.003	0.842
Ruhango	0.544	0.194	0.435	0.017		0.120	0.092	0.012	0.016
Rulindo	0.718	0.564	0.174	0.386		0.468	0.456	0.004	0.116
Rusizi	0.715	0.559	0.254	0.361		0.754	0.549	0.096	0.549
Rutsiro	0.935	0.805	0.264	0.687		0.877	0.835	0.005	0.599
Rwamagana	0.701	0.027	0.683	0.016		0.077	0.012	0.056	0.008
